# On the spider genus *Amaurobius* (Araneae, Amaurobiidae) in India and Nepal

**DOI:** 10.3897/zookeys.168.2352

**Published:** 2011-01-31

**Authors:** Yuri M. Marusik, Francesco Ballarin, Mikhail M. Omelko

**Affiliations:** 1 Institute for Biological Problems of the North of the Russian Academy of Sciences, Portovaya Str. 18, 685000 Magadan, Russia; 2 Zoological Museum, University of Turku, FI-20014, Turku, Finland; 3 Museo Civico di Storia Naturale di Verona, Lungadige Porta Vittoria, 9 – 37129, Verona, Italy; 4 Far Eastern Federal University, Sukhanova, 8, Vladivostok 690950 Russia; 5 Gornotaezhnaya Station FEB RAS, Gornotaezhnoe Vil., Ussuriyski Dist., Primorski Krai 692533 Russia

**Keywords:** Amaurobiidae, Titanoecidae, India, Asia, key, new combination

## Abstract

A new species, *Amaurobius koponeni* **sp. n.**, is described from Himachal Pradesh on the basis of a male specimen. A key to all five genera of Amaurobiidae that occur in Asia is provided. Four species from India and Nepal incorrectly assigned to *Amaurobius* are transferred to three genera of Titanoecidae: *Anuvinda milloti* (Hubert, 1973), **comb. n.**, *Pandava andhraca* (Patel & Reddy, 1990), **comb. n.**, *Pandava nathabhaii* (Patel & Patel, 1975), **comb. n.**, and *Titanoeca sharmai* (Bastawade, 2008), **comb. n.**

## Introduction

*Amaurobius* C.L. Koch, 1837, is a rather large genus with 68 valid species names (Platnick 2011). It has a primarily Holarctic distribution. Only six species of this genus have been recorded outside of this region: *Amaurobius andhracus* Patel & Reddy, 1990, *Amaurobius nathabhaii* Patel & Patel, 1975, *Amaurobius sharmai* Bastawade, 2008 (all in India), *Amaurobius thoracicus* Mello-Leitão, 1945 (Argentina), *Amaurobius tristis* L. Koch, 1875 (Eritrea) and *Amaurobius yanoianus* Nakatsudi, 1943 (Micronesia). Only the first two of these are known from both sexes. The other species are known either from female or by juvenile (*Amaurobius thoracicus*) specimens and appear to have been incorrectly assigned to the genus and even possibly to the family.

Recently, we found a specimen belonging to *Amaurobius* from northern India and whilst trying to identified it, we checked all species (descriptions) known from India and Nepal. The study of these descriptions revealed that all the so-called amaurobiid species were misplaced and actually belong to Titanoecidae, and at least *Amaurobius sharmai* is likely to belong to *Titanoeca* Thorell, 1870. Another *Amaurobius*, *Amaurobius milloti* Hubert, 1973, known from Nepal also seems to have been misplaced and belongs to the titanoecid genus *Anuvinda* Lehtinen, 1967.

The aims of this paper are to describe a new species of *Amaurobius*, to provide a key to the amaurobiid genera that occur in Asia, and to transfer the misplaced species to Titanoecidae.

## Material and methods

Microphotographs were made with an Olympus Camedia E-520 camera attached to an Olympus SZX16 stereomicroscope at the Zoological Museum, University of Turku. Digital images were montaged using “CombineZP” image stacking software. Photographs were taken in paraffin-based dishes using different sized holes to keep the samples in the required position. The holotype of the new species is preserved in the collections of the Museo Civico di Storia Naturale di Verona, Italy (MSNV). Comparative specimens illustrated are from Russia, Kunashir Island (*Cybaeopsis* and *Callobius*) and Magadan Area (*Arctobius*) and from Finland (female of *Amaurobius fenestralis*).

All measurements are in millimetres.

## Taxonomic survey

To date, five genera of amaurobiid spiders have been recorded from Asia east of the Caucasus: *Amaurobius* C.L. Koch, 1837 (India), *Arctobius* Lehtinen, 1967 (the whole of Siberia south to Mongolia), *Callobius* Chamberlin, 1947 (Far East), *Cybaeopsis* Strand, 1907 (Far East) and *Taira* Lehtinen, 1967 (Far East and South East). All genera except *Arctobius* (subfamily Arctobiinae) belong to the nominative subfamily Amaurobiinae. *Arctobius* differs distinctly from all other amaurobiids by colour, markings and eye arrangement. Amaurobiinae genera can be relatively easily distinguished by the structure of the palp in males and the epigyne in females. *Taira* has a reduced or absent retrolateral tibial apophysis (cf. Zhang et al. 2008; Wang et al. 2010). *Callobius* and *Cybaeopsis* differ from other genera by possessing a strong and long dorsal tibial apophysis, and in having the epigyne divided into two lobes. In *Callobius* the epigyne has a median lobe which is absent in *Cybaeopsis* (Ubick 2005). *Amaurobius* has a dorsal tibial apophysis without long extensions and the epigyne is transverse and undivided.

### Key to the genera of Amaurobiidae found in Asia

Females of *Amaurobius* and *Taira* have no distinct morphological differences (cf. Zhang et al. 2008)

**Table:** 

1	Anterior median eyes equidistant from each other and anterior lateral eyes; abdomen with dark median band (*Mb*, Fig. 9), male palpal tibia without dorsal apophysis (Fig. 11), epigyne with strongly sclerotized median part of median plate (Fig. 10). Occurs in the whole of Siberia south to Mongolia	*Arctobius agelenoides* (Emerton, 1919)
–	Anterior median eyes closer to each other than to lateral eyes; median band not developed or developed only in anterior half (Fig. 1), male palpal tibia with distinct dorsal apophysis (Figs 2, 4–8, 13, 15), epigyne bilobate (Figs 12, 14) or with weakly sclerotized median plate (Fig. 16)	2
2	Dorsal tibial apophysis long, partly overlying cymbium (Figs 13, 15), epigyne bilobate (Figs 12, 14)	3
–	Dorsal tibial apophysis massive (Figs 2, 4–8), but not long, not overlying cymbium, epigyne with median plate, not bilobate (Fig. 16)	4
3	Dorsal tibial apophysis with three branches (*Bd*), retrolateral tibial apophysis (*Ra*) bilobate on the top (Fig. 13), epigyne without median lobe (Fig. 12). Occurs in Far East Asia.	*Cybaeopsis typicus* Strand, 1907
–	Dorsal tibial apophysis not subdivided (Fig. 15), retrolateral tibial apophysis (*Ra*) elongate dorsally; epigyne with median lobe (Fig. 14). Occurs in Far East Asia	*Callobius*
4	Retrolateral tibial apophysis large (Figs 2–5, 8); tegular apophysis located near the base of median apophysis. Epigyne with transverse lobe or fovea (Fig. 16). Occurs in northern India.	*Amaurobius*
–	Retrolateral tibial apophysis small (knob-like) or absent; tegular apophysis originates near base of embolus. Occurs in Japan and China	*Taira*

**Comments**. *Cybaeopsis* Strand, 1907 is a relatively small genus with 11 species, of which only one, *Cybaeopsis typica* Strand, 1907, occurs in Japan and the Russian Far East (Sakhalin, South and Middle Kuril Islands (Platnick 2011). The remaining 10 species are restricted to the Nearctic. *Callobius* Chamberlin, 1947 is a rather large genus with 30 species distributed in the Western Palaearctic, Far East Asia (Japan, Korea and Kunashir Island) and the Nearctic. Only three species are known from Asia: *Cybaeopsis hokkaido* Leech, 1971 (Hokkaido and Kunashir Islands), *Cybaeopsis koreanus*(Paik, 1966) (Korea) and *Cybaeopsis akushimensis* Okumura, 2010 (Japan) (cf. Platnick 2011; Marusik and Kovblyuk 2011). *Taira* Lehtinen, 1967 is a relatively small genus with 11 species restricted to China and Japan (Platnick 2011).

#### Amaurobius koponeni sp. n.

urn:lsid:zoobank.org:author:36297992-069D-47FC-9B60-9B26EB2C7698

http://species-id.net/wiki/Amaurobius_koponeni

Figs 1 –8

##### Type material.

Holotype ♂ (MSNV), India, Uttar Pradesh, Farrukhabad District, Kaimganj City [=27.550°N, 79.332°E], 23.03.2003 (F. Abrescia).

##### Etymology. 

The species is named after our friend and colleague Seppo Koponen (Turku, Finland).

##### Diagnosis.

The new species differs distinctly from other congeners by the shape of the tibial apophysis and the median apophysis.

##### Description.

**Figures 1–2.**
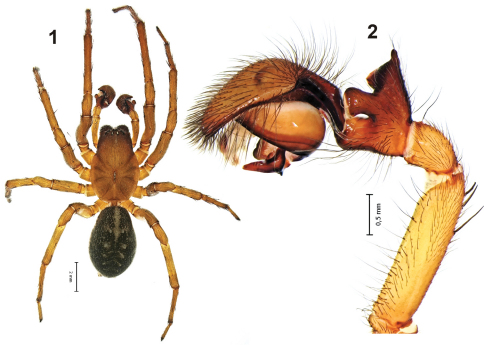
Male of *Amaurobius koponeni* sp. n. **1** habitus **2** left palp, retrolateral.

Total length 9.8. Carapace length 4.95, width 3.4. Habitus as in Fig. 1. Carapace light brown with dorsal darker radiating strips, fovea and eye region dark brown. Chelicerae dark, swollen in front with four posterior and five anterior teeth.

Legs light brownish without rings, tarsi with three claws, scopula and claws tufts absent. Calamistrum about 1/3 of metatarsus length.

Length of leg segments:

Leg spination:

Sternum without pattern, same colour as carapace. Abdomen dark grey with dorsal and ventral pattern, cribellum clearly visible.

**Figures 3–8.**
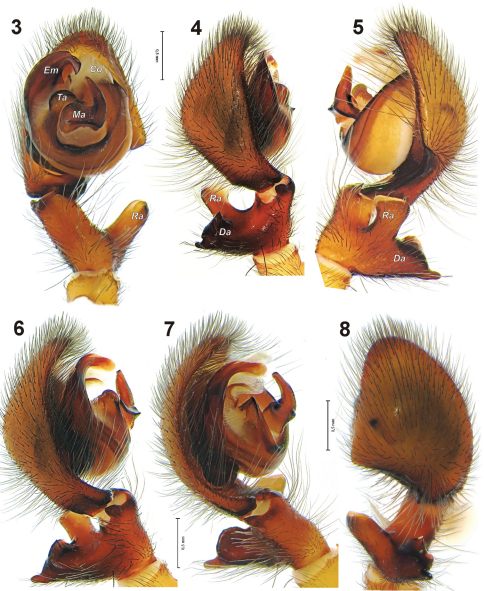
Left palp of *Amaurobius koponeni* sp. n. **3** ventral **4, 6–7** prolateral, different aspects showing the shape of the complex dorsal tibial apophysis **5** prolateral **8** dorsal. Abbreviations: ***Da*** dorsal tibial apophysis, ***Em*** embolus, ***Ma*** median apophysis, ***Ra*** retrolateral tibial apophysis, ***Ta*** tegular apophysis.

Palp as in Figs 2–8, tibia with large square-shaped retrolateral tibial apophysis (*Ra*) originating near the base of the tibia and almost as long as the tibia In ventral view the tibia and *Ra* have a V-shape; dorsal tibial apophysis (*Da*) large and massive, its length almost twice as long as the diameter of the tibia; intermediate apophysis not developed (or fused with *Da*). Retrobasal part of cymbium with long fold of about ½ of the cymbium. Median apophysis (*Ma*) massive, located in the center of the tegulum, basal half of it horizontal and terminal part almost vertical; conductor wide, as wide as basal half of *Ma*; embolus (*Em*) sharply pointed.

##### Distribution.

The new species is known from the type locality only, the area near the city of Kaimganj in Uttar Pradesh, India.

### Notes on species misplaced in Amaurobius

As mentioned above, three species of *Amaurobius* (*Amaurobius andhracus* Patel & Reddy, 1990, *Amaurobius nathabhaii* Patel & Patel, 1975 and *Amaurobius sharmai* Bastawade, 2008) have been recorded from India (Platnick 2011) and one more species is known from Nepal (*Amaurobius milloti* Hubert, 1973). All these species were misplaced in Amaurobiidae and actually belong in Titanoecidae. It is worth mentioning that recently one more species, *Amaurobius indicus* Bastawade, 2002 was described in the genus. Again, this was misplaced and it actually belongs in Corinnidae. It would appear that the Indian authors have an incorrect concept of the genus and of the family in general.

The genus *Pandava* was revised by Almeida-Silva et al. (2010) and five species were described as new to science. Of these, four species were described from India: *Pandava shiva* Almeida-Silva et al., 2010, *Pandava ganga* Almeida-Silva et al., 2010, *Pandava kama* Almeida-Silva et al., 2010 and *Pandava ganesha* Almeida-Silva et al., 2010 (Fig. 17). Therefore, it is possible that some of their new names may be synonyms of Indian “*Amaurobius*”.

**Figures 9–16.**
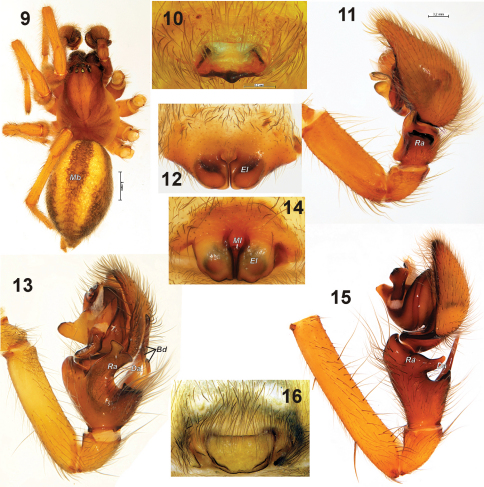
Habitus and copulatory organs of *Arctobius agelenoides* (**9–11**, from Magadan Area), *Cybaeopsis typicus* (**12–13**, from Kunashir Island), *Callobius hokkaido* (**14–15**, from Kunashir Island) and *Amaurobius fenestralis* (**16**, from South Finland). **9** habitus **10, 12, 14, 16** epigyne, ventral **11, 13, 15** left palp retrolateral. Abbreviations: ***Bd***branches of dorsal tibial apophysis, ***Da*** dorsal tibial apophysis, ***El*** lateral lobe of epigyne, ***Ml***median lobe of epigyne ***Ra*** retrolateral tibial apophysis.

#### Anuvinda milloti

(Hubert, 1973) comb. n.

http://species-id.net/wiki/Anuvinda_milloti

Amaurobius milloti Hubert, 1973a: 676, f. 1-6 (♂♀).

##### Comments.

**Figure 17.**
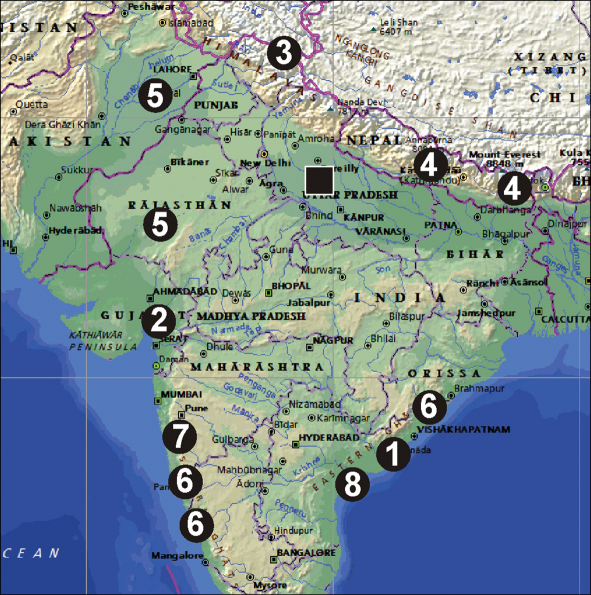
Distribution of *Amaurobius koponeni* sp. n. (square), four species transferred here into Titanoecidae: *Pandava andhraca* **1** *Pandava nathabhaii* **2** *Titanoeca sharmai* **3** *Anuvinda milloti* **4** and four *Pandava* species recently described from India: *Pandava shiva* **5** *Pandava ganga* **6** *Pandava kama* **7** and *Pandava ganesha* **8**.

This species is perfectly described from central and eastern Nepal (Fig. 17). Judging from the structure of the male palp, and particularly the modified patella, it undoubtedly belongs to *Anuvinda* Lehntinen, 1967, the type species of which, *Anuvinda escheri* (Reimoser, 1934) was recently well redescribed on the basis of both sexes by Almeida-Silva et al. (2009). Judging from the diagnosis and figures of the copulatory organs of *Anuvinda escheri* (Reimoser, 1934), it is very likely that the two names should be synonymized. An additional argument which supports their probable synonymy is the distribution of both species. *Anuvinda escheri* is known from central India (type locality), Thailand, Laos and southern China (Yunnan) and *Anuvinda milloti* has been recorded from several localities in central and eastern Nepal.

#### Pandava andhraca

(Patel & Reddy, 1990) comb. n.

http://species-id.net/wiki/Pandava_andhraca

Amaurobius andhracus Patel & Reddy, 1990: 41, f. 1a–h (♂♀).

##### Comments.

This species was described on the basis of both sexes from Andhra Pradesh (Fig. 17), but the description and figures are of poor quality. Judging from the figures of the male palp this species belongs in Titanoecidae. Judging from the colour, shape of the epigyne and its distribution, the species belongs to *Pandava*, a titanoecid genus restricted to India, Sri Lanka, southern China, Myanmar and Thailand. The type species of the genus has a broader distribution. Judging from the shape of the epigyne, this species may be a junior synonym of *Pandava laminata* (Thorell, 1878), the type species of the genus, known from East Africa to the Philippines and Marquesas Islands.

#### Pandava nathabhaii

(Patel & Patel, 1975) comb. n.

http://species-id.net/wiki/Pandava_nathabhaii

Amaurobius nathabhaii Patel & Patel, 1975: 801, f. 1a–c (♀).

##### Comments.

This species was described on the basis of the female sex from Gujarat (Fig. 17), but the description and figures are of very poor quality. This species is placed in Titanoecidae because the other Indian species placed in *Amaurobius* belong to Titanoecidae. It is transferred to *Pandava* because of its southern distribution.

#### Titanoeca sharmai

(Bastawade, 2008) comb.n.

http://species-id.net/wiki/Titanoeca_sharmai

Amaurobius sharmai Bastawade, 2008: 40, f. 1-12 (♂♀).

##### Comments.

This species was described from the northeastern region of Himachal Pradesh, India (Fig. 17) on the basis of both sexes, but the figures and description are of rather poorly quality. The figures provided by the author, namely the tibial and metatarsal spines on the legs in males and the structure of the palp, leaves no doubt that the species belongs in Titanoecidae. Although there are three titanoecid genera in India, judging from the locality, high elevation, and the unmodified male palpal patella, *Amaurobius sharmai* must be placed in *Titanoeca*. It is worth mentioning that this species may be a junior synonym of *Titanoeca intermedia* Caporiacco, 1934 (species incorrectly synonymized with *Titanoeca flavicoma* L. Koch, 1872), which was described from territories now belonging to northeastern Pakistan and from northern India (Jammu & Kashmir).

## Supplementary Material

XML Treatment for Amaurobius koponeni

XML Treatment for Anuvinda milloti

XML Treatment for Pandava andhraca

XML Treatment for Pandava nathabhaii

XML Treatment for Titanoeca sharmai

## References

[B1] Almeida-Silva LM Brescovit AD Griswold CE (2009) On the poorly known genus *Anuvinda* Lehtinen, 1967 (Araneae: Titanoecidae). Zootaxa 2266: 61-68

[B2] Almeida-Silva LM Griswold CE Brescovit AD (2010) Revision of the Asian spider genus *Pandava* Lehtinen (Araneae: Titanoecidae): description of five new species and first record of Titanoecidae from Africa. Zootaxa 2630: 30-56

[B3] Bastawade DB Borkar M (2008) Arachnida (orders Scorpiones, Uropygi, Amblypygi, Araneae and Phalangida). In: Fauna of Goa, State Fauna Series. Zoological Survey of India 16: 211-242

[B4] Bastawade DB (2002) Three new species from the spider families Amaurobiidae, Thomisidae and Salticidae (Araneae: Arachnida) from India. The Journal of the Bombay Natural History Society 99: 274-281

[B5] Hubert M (1973) Araignées du Népal 1. Description d’*Amaurobius milloti* sp. n. (Amaurobiidae) et répartition de *Psechrus himalayanus* Sim. (Psechridae). Bulletin du Museum National d’Histoire Naturelle - Paris (Zool.) 97: 675-68215859950

[B6] Marusik YM Kovblyuk MM (2011) Spiders of Siberia and Russian Far East. KMK Scientific Press, Moscow. 344 pp. [in Russian]

[B7] Patel BH Patel HK (1975) A new record of the family Amaurobiidae (Arachnida: Araneae) from India. The Journal of the Bombay Natural History Society 72: 800-803

[B8] Patel BH Reddy TS (1990) A new species of *Amaurobius* Koch (Araneae: Amaurobiidae) from coastal Andhra Pradesh, India. Entomon 15: 41-43

[B9] Platnick NI (2011)The World Spider Catalog, Version 12.0. American Museum of Natural History, New York. At http://research.amnh.org/iz/spiders/catalog/ [accessed 01 November 2011]

[B10] Ubick D (2005) New genera and species of cribellate coelotine spiders from California (Araneae: Amaurobiidae). Proceedings of California Academy of Sciences 56: 305-336

[B11] Wang XP Jäger P Zhang ZS (2010) The genus *Taira*, with notes on tibial apophyses and descriptions of three new species. The Journal of Arachnology 38: 57-72 doi: 10.1636/A09-19.1

[B12] Zhang ZS Zhu MS Song DX (2008) Revision of the spider genus *Taira* (Araneae, Amaurobiidae, Amaurobiinae). The Journal of Arachnology 36: 502-512 doi: 10.1636/H07-49.1

